# Experimental Identification of *Actinobacillus pleuropneumoniae* Strains L20 and JL03 Heptosyltransferases, Evidence for a New Heptosyltransferase Signature Sequence

**DOI:** 10.1371/journal.pone.0055546

**Published:** 2013-01-30

**Authors:** Susana Merino, Yuriy A. Knirel, Miguel Regué, Juan M. Tomás

**Affiliations:** 1 Departamento de Microbiología, Facultad de Biología, Universidad de Barcelona, Barcelona, Spain; 2 N. D. Zelinsky Institute of Organic Chemistry, Russian Academy of Sciences, Moscow, Russia; 3 Departamento de Microbiología y Parasitología Sanitarias, Facultad de Farmacia, Universidad de Barcelona, Barcelona, Spain; Cornell University, United States of America

## Abstract

We experimentally identified the activities of six predicted heptosyltransferases in *Actinobacillus pleuropneumoniae* genome serotype 5b strain L20 and serotype 3 strain JL03. The initial identification was based on a bioinformatic analysis of the amino acid similarity between these putative heptosyltrasferases with others of known function from enteric bacteria and *Aeromonas*. The putative functions of all the *Actinobacillus pleuropneumoniae* heptosyltrasferases were determined by using surrogate LPS acceptor molecules from well-defined *A. hydrophyla* AH-3 and *A. salmonicida* A450 mutants. Our results show that heptosyltransferases APL_0981 and APJL_1001 are responsible for the transfer of the terminal outer core D-*glycero*-D-*manno*-heptose (D,D-Hep) residue although they are not currently included in the CAZY glycosyltransferase 9 family. The WahF heptosyltransferase group signature sequence [S(T/S)(GA)XXH] differs from the heptosyltransferases consensus signature sequence [D(TS)(GA)XXH], because of the substitution of D^261^ for S^261^, being unique.

## Introduction

*Actinobacillus pleuropneumoniae* is a non-motile Gram-negative bacterium causing porcine pleuropneumonia, a highly contagious respiratory disease transmitted through aerosols or close contact with infected animals including asymptomatic carriers [1]. This disease is often fatal and characterized by hemorrhagic, fibrinous and necrotic lung lesions; the clinical features ranging from acute to chronic [2], and it is an important cause economic losses worldwide in the porcine industry [2].

To date, two biovars have been described on the basis of NAD growth dependence; and fifteen serotypes based on capsular antigens [3], [4]; the disease can be caused by any serotype, although differences in virulence have been described [5], [6].

Studies aimed at the identification of *A. pleuropneumoniae* virulence factors showed that, as in other pathogenic bacteria, there is an array of these factors. However, the most relevant one appears to be the Apx toxins [5]. Other possible virulence factors include the capsule [7], [8], [9], outer membrane proteins involved in iron uptake [1], [10], [11], and lipopolysaccharides (LPS) [12], [13]. Biofilm formation [14], autotransporter adhesion[15], and autotransporter protease synthesis [16] have been also described to contribute to the pathogenicity of this bacterium.

The LPS appears to play a role in virulence in different stages of the *A. pleuropneumoniae* infection, including adhesions to lower respiratory tract, induction of lesions, and persistence in the upper respiratory tract reviewed in [17]. In addition the LPS molecules appear to interact with ApxI and ApxII toxins [18].

**Figure 1:**
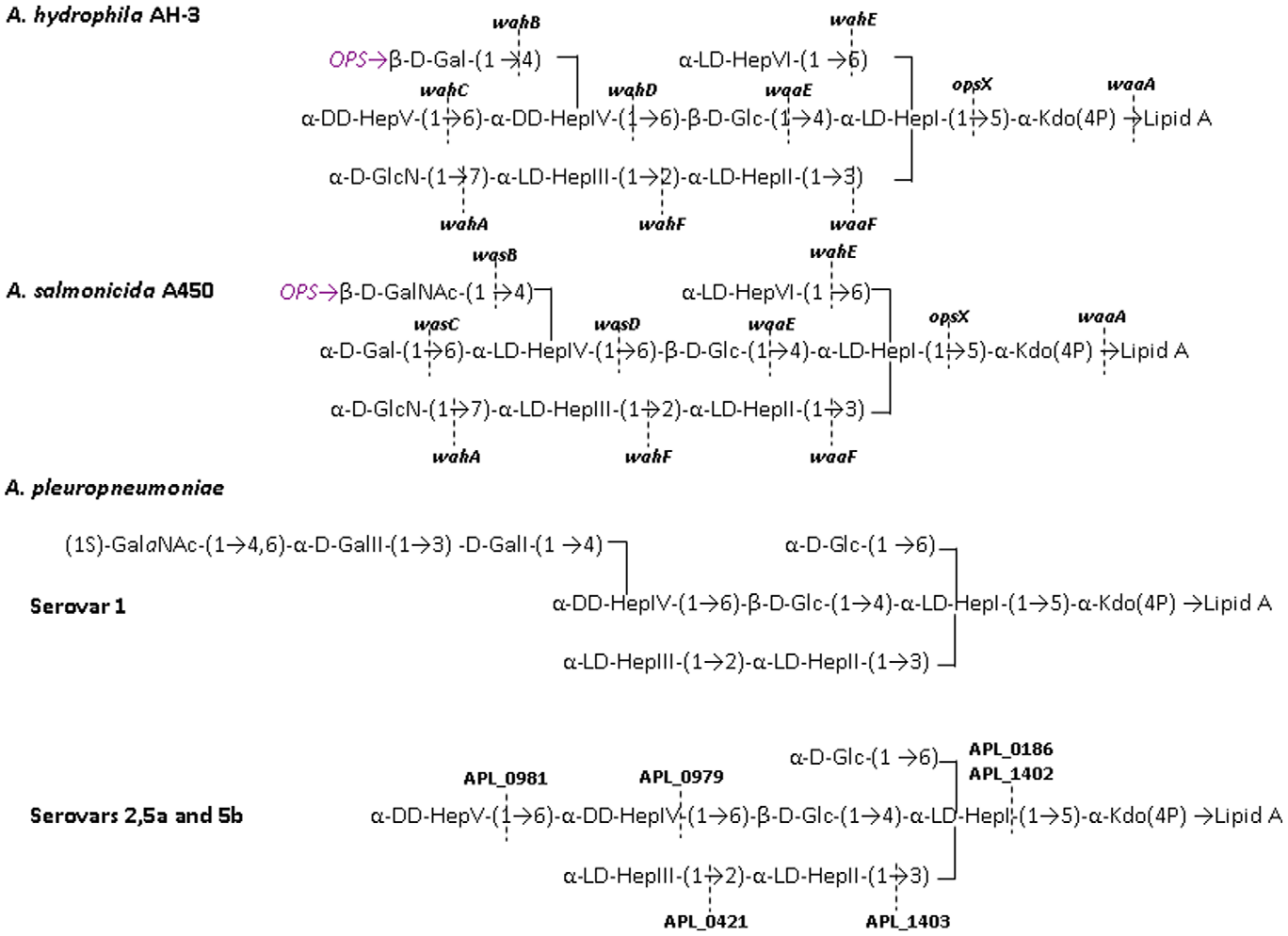
LPS core chemical structures for *A. hydrophila* [**24**], *A. salmonicida* [**33**], and *A. pleuropneumoniae* serotypes 1, 5a, and 5b [**22**]. The genes involved in core LPS biosynthesis are shown for *A. hydrophila* and *A. salmonicida* [25], [33].

The LPS of *A. pleuropneumoniae* consist of three domains: an endotoxic glycolipid (lipid A), an O-polysaccharide chain (O-PS or O-antigen), and an intervening core oligosaccharide (core-OS). The structures the O-PS present in fourteen out of the fifteen capsular serotypes have been determined [19], [20], [21]. By contrast, the core LPS structure has been elucidated only for representative strains of *A. pleuropneumoniae* i.e., serotypes 1 (strain 4074), 2 (strain 4226), 5a (strain K17), and 5b (strain L20) [22]. All these strains share a common heptasaccharide, but differ in substitutions at D,D-HepIV residue (Figure 1). The common heptasaccharide includes three L-*glycero*-D-*manno*-heptose (L,D-Hep) and one D-*glycero*-D-*manno* heptose (D,D-Hep) residues. Relative to serotype 1, an additional D,D-HepV residue was found in serotypes 2, 5a, and 5b (Figure 1).

Until now only one *A. pleuropneumoniae* heptosyltransferase was identified for a strain belonging to the serotype 1 [23]. Since the common heptasaccharide present in the core OS of the *A. pleuropneumoniae* studied strains is highly similar to the core OS of *A. hydrophila* [24] (Figure 1) we decided to use well defined core mutants of *A- hydrophila* AH-3 [25] producing surrogate acceptor LPS molecules to identify the heptosyltransferases involved in the core OS biosynthesis of genome strains belonging to serotypes 3 and 5b.

## Results

### Bioinformatic and phylogenetic analysis

**Figure 2:**
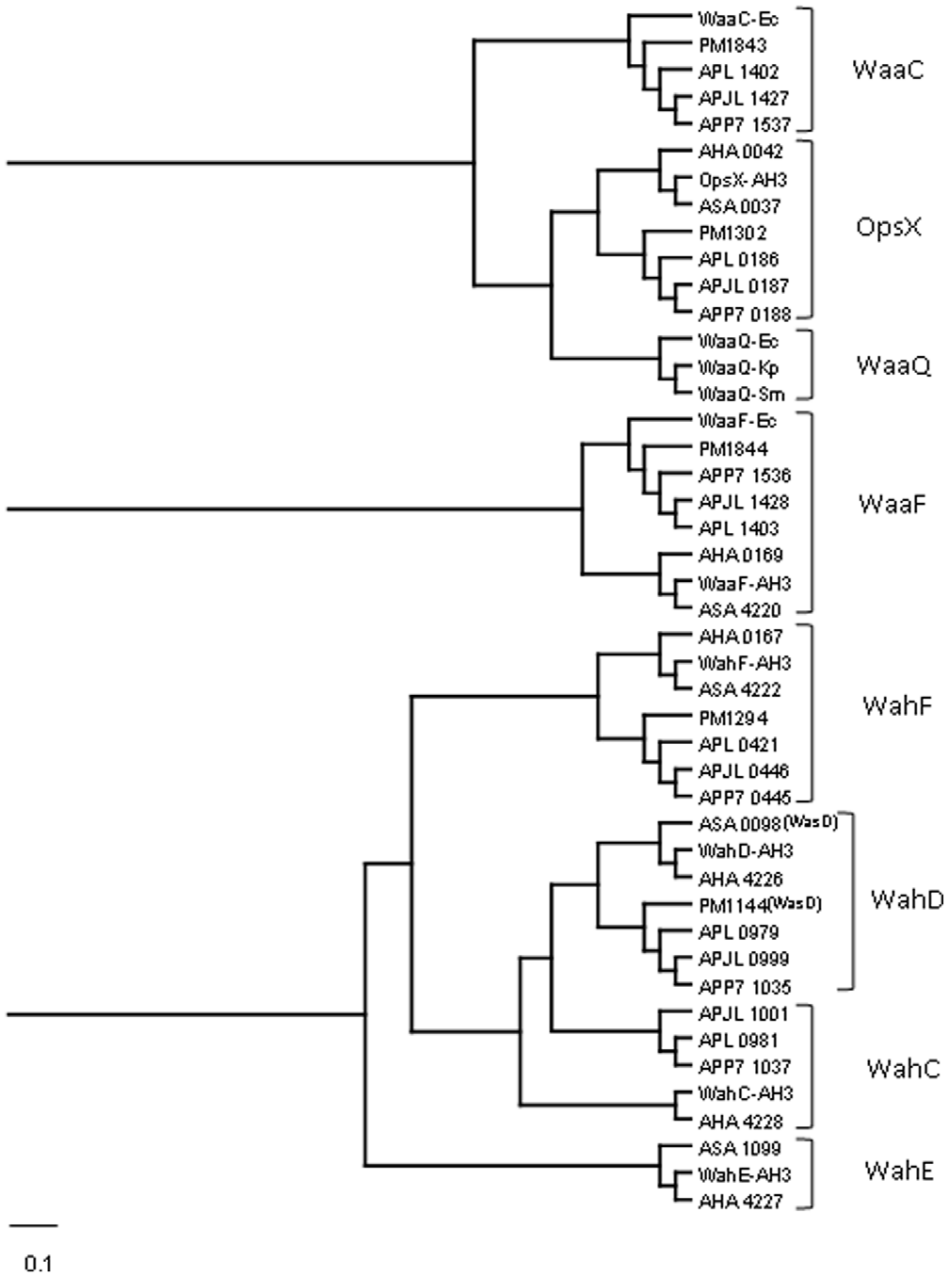
Cladogram obtained running the phylogenetic inference software Protpars from the PHYLIP package version 3.5c as indicated in Materials and Methods section. The separation and the large of the lanes are relative to the similarity degree according to the program used (the scale bar indicates an evolutionary distance of 0.1 aminoacid substitutions per position).The cladogram shows the similarity relationship among putative heptosyltransferases from *A. pleunopneumoniae* strains L20, JL03, and AP76 (APL, APJL, and APP7, respectively), *A. hydrophila* AH3 and ATCC7966^T^ strains (AH-3 and AHA, respectively), *A. salmonicida* A450 strain (ASA), *E. coli* (Ec), *K. pneumoniae* (Kp), *S. marcescens* (Sm), and *P. multocida* (PM).

The core LPS structures of *A. pleuropneumoniae* studied strains contain between four and five heptose residues (Figure 1) requiring an equal number of heptosyltransferases. These heptosyltransferases were predicted on the basis of the nature of the substrate heptose, either L,D-Hep or D,D-Hep, and the linkage to the corresponding substrate core residue. In order to attribute putative functions we performed a bioinformatic analysis based on the alignment (Clustal W) of heptosyltransferases whose function is experimentally proved with those of *A. pleuropneumoniae* strains L20 (serotype 5b) [26], JL03 (serotype 3) [27], and AP76 (serotype 7) [28]. After this alignment, we performed a phylogenetic analysis using the same heptosyltransferases whose function is experimentally proved and the ones obtained with *A. pleuropneumoniae* strains. The cladogram obtained is shown in Figure 2 as an indicative of the similarity degree among these proteins.

In the Carbohydrate-Active EnZymes database (CAZy) (http://www.cazy.org) five glycosyltransferases (APL_0186, APL_0421, APL_0979, APL_1402, and APL_1403) belonging to family GT9, that includes known heptosyltransferases, are reported for the genome strain L20 (serotype 5b) [26]. Similarly five homologues of these heptosyltransferases are found in *A. pleuropneumoniae* genome strains JL03 (serotype 3) [27] and AP76 (serotype 7) [28]. The presence of these heptosyltransferases is in apparent agreement with the five heptose residues present in the L20 strain core LPS (3 L,D-Hep and 2 D,D-Hep) [23] (Figure 1). However, it should be noted that APL_0186 (OpsX) and APL_1402 (WaaC) correspond to two different versions of heptosyltransferase I.

OpsX and WaaC transfer the first L,D-Hep residue to phosphorylated and unphosphorylated Kdo, respectively [25], [29], [30]. Indeed enzymes (APL_0904 and APJL_0916) for the phosphorylation at the 4-OH position of Kdo are also present in strain L20 or JL03, respectively. As expected these two heptosyltransferases, on the basis of amino acid similarity, cluster in two different branches when analyzed using Clustal W (Figure 2). *Aeromonas hydrophila* strains ATCC 7966 and AH-3 and *A. salmonicida* A450 contain only the OpsX version (AHA_0042, OpsX-AH3 and ASA-0037) and as expected their core LPS oligosaccharides only contain phosphorylated Kdo [24]. The genome strain Pm70 of *Pasteurella multocida* contains both versions of heptosyltransferase I and analysis of the core LPS of strains Pm70 and VP161has shown that their core LPS contains two major oligosaccharides only differing in their Kdo phosphorylation [31], [32]. No phosphorylated Kdo was reported in the core LPS of the four *A. pleuropneumoniae* strains studied [32] probably because in this study the core fraction was obtained by acid hydrolysis that could remove the phosphate from Kdo.

The same Clustal W analysis shows that known heptosyltransferases from *Aeromonas hydrophyla* and *salmonicida* transferring L,D-Hep II (WaaF) and L,D-Hep III (WahF) cluster with putative heptosyltransferases from *A. pleuropneumoniae* L20 (APL_1403 and APL_0421), and *Pasteurella multocida* Pm70 (Figure 2) in agreement with the presence of a common trisaccharide of L,D-Hep in the inner core of these bacteria (Figure 1).

A fourth cluster encompasses *A. hydrophila* WahD (AHA_4226) and *A. salmonicida* WasD (ASA_0098) with *A. pleuropneumoniae* APL_0979 and *P. multocida* PM1144. WahD and WasD transfer a D,D-Hep and L,D-Hep residue attached by an α 1,6 linkage to the β-D-Glc residue in *A. hydrophila* and *A. salmonicida*, respectively [25], [33]. Thus, they should recognize a very similar LPS substrate only differing in the donor sugar, either ADP-D,D-Hep or ADP-L,D-Hep. The presence of the disaccharide α-D.D-Hep (1,6) β-D-Glc and α-L.D-Hep (1,6) β-D-Glc in *A. pleuropneumoniae* (serotypes 1, 2, 5a and 5b) and *P. multocida*, respectively, suggest that APL_0970 and PM1144 are homologues of WahD and WasD.

Two proximal clusters includes WahC from *A. hydrophila* and APL_0981, APLJ_1001, and APP7_1037 from *A. pleuropneumoniae* genomic strains L20 (serotype 5b), J03 (serotype 3) and AP76 (serotype 7). WahC has been shown to be the heptosyltransferase responsible for the transfer of the terminal D,D-Hep residue of the *A. hydrophila* AH-3 core LPS [25] (Figure 1) and although their counterparts from *A. pleuropneumoniae* are actually included in a non-classified group of glycosyltransferases (GTnc, http://www.cazy.org) the present analysis could suggest a common function for all these proteins.

As a control for this analysis we used the WahE proteins from *A. hydrophila* and *salmonicida* involved in the transfer of a L,D-Hep residue by an α 1,6 linkage to L,D-Hep I [25], [33] (Figure 1). As shown in Figure 2 no similar proteins from *A. pleuropneumoniae* or *P. multocida* fall in this cluster.

### Functional identification of the two versions of *A. pleuropneumoniae* heptosyltransferase I

In order to unambiguously identify the functions of the above putative heptosyltransferases we decided to use LPS from several *A. hydrophila* AH-3 strain mutants as a source of surrogate LPS acceptor molecules. These mutants were previously constructed by either Km^R^ insertion or internal in-frame deletion in such a way to avoid polar effects on downstream genes [25].

**Figure 3:**
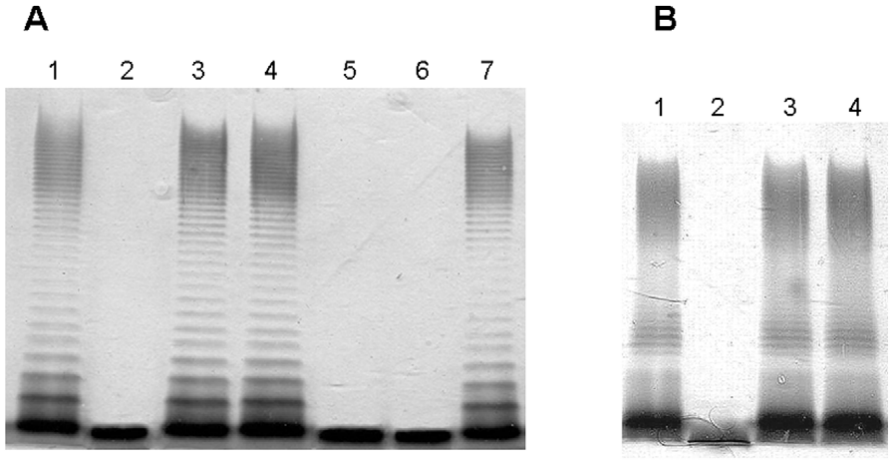
LPS analysis by SDS-PAGE. (**A**) LPS samples from *A. hydrophila* AH-3 (lanes 1 and 7), AH-3Δ*opsX* (lane 2), AH-3Δ*opsX* (pBAD33-Gm-APL_0186) (lane 3), AH-3Δ*opsX* (pBAD33-Gm-APJL_0187) (lane 4), AH-3Δ*opsX* (pBAD33-Gm-APL_1402) (lane 5), and AH-3Δ*opsX* (pBAD33-Gm-APJL_1427) (lane 6). (**B**) LPS samples from *K. pneumoniae* 52145 (lane 1), 52145Δ*waaC* (lane 2), 52145Δ*waaC* (pBAD33-Gm-APL_1402) (lane 3), and 52145Δ*waaC* (pBAD33-Gm-APJL_1427) (lane 4).

First, we established the function of APL_0186 by its expression under the control of the P_ARA_ promoter (pBAD33-Gm-APL_0186). The core LPS from *A. hydrophila* AH-3Δ*opsX* mutant only contains a phosphorylated Kdo residue and is devoid of O-antigen [25]. The pBAD33-Gm-APL_0186 was introduced into *A. hydrophila* AH-3Δ*opsX* mutant and LPS from this strain was analyzed by SDS-PAGE after growth in inducing conditions. The APL_0186 expression plasmid fully complemented the *opsX* mutation leading to the production of full length LPS including O-antigen (Figure 3A, lane 3). The same result was obtained when using APJL_0187 expression plasmid from *A. pleuropneumoniae* strain JL03 (serotype 3) (Figure 3A, lane 4). These results strongly suggest that APL_0186 and APJL_0187 correspond to the OpsX version of heptosyltransferase I. By contrast, neither APL_1402 nor APJL_1427 were able to modify the LPS migration pattern of *A. hydrophila* AH-3Δ*opsX* mutant (Figure 3A, lanes 5 and 6), suggesting that they could correspond to the WaaC version of heptosyltransferase I. In agreement with this hypothesis, expression of APL_1402 and APJL_1427 in *K. pneumoniae* 52145Δ*waaC* [34] were able to restore the full length *K. pneumoniae* 52145 core LPS and O-antigen production (Figure 3B, lanes 3 and 4). These results establish that *A. pleuropneumoniae* contains the two versions of heptosyltransferase I.

### Heptosyltransferases II and III

**Figure 4:**
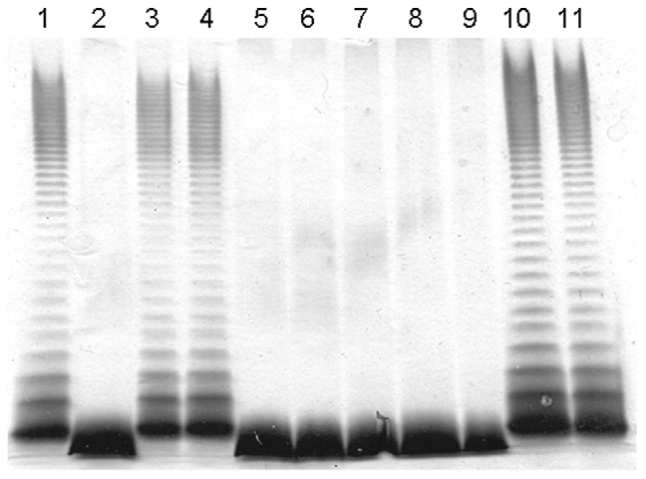
SDS-PAGE analysis of LPS prepared from *A. hydrophila* AH-3 (lane 1), AH-3 Δ***waaF*** **(lane 2), AH-3**Δ***waaF*** **(pBAD33-Gm-APL_1403) (lane 3), AH-3**Δ***waaF*** **(pBAD33-Gm-APJL_1428) (lane 4), AH-3**Δ***waaF*** **(pBAD33-Gm-APL_0421) (lane 5), AH-3**Δ***waaF*** **(pBAD33-Gm-APJL_0446) (lane 6), AH-3**Δ***wahF*** **(lane 7), AH-3**Δ***wahF*** **(pBAD33-Gm-APL_1403) (lane 8), AH-3**Δ***wahF*** **(pBAD33-Gm-APJL_1428) (lane 9), AH-3**Δ***wahF*** **(pBAD33-Gm-APL_0421) (lane 10), and AH-3**Δ***wahF*** **(pBAD33-Gm- APJL_0446) (lane 11).**

The LPS from *A. hydrophila* AH-3Δ*waaF* is devoid of O-antigen and contains a trisaccharide core [α-L,D-Hep-(1→6)-α-L,D-Hep-(1→5)-Kdo-P] [25]. This core structure is similar to the one expected from a *A. pleuropneumoniae waaF* mutant [α-L,D-Hep-(1→5)-Kdo-P] and it was expected that the putative WaaF enzymes APL_1403 and APJL_1428 would be able to recognized the *A. hydrophila* AH-3Δ*waaF* core. As expected, expression of APL_1403 (pBAD33-Gm-APL_1403) or APJL_1428 (pBAD33-Gm-APJL_1428) into *A. hydrophila* AH-3Δ*waaF* resulted in the production of full length *A. hydrophila* AH-3 core including O-antigen (Figure 4, lanes 3 and 4). By contrast, expression of APL_0421 or APJL_0446 in this genetic background did not modify the mutant A. *hydrophila* AH-3Δ*waaF* core LPS (Figure 4, lanes 5 and 6).

On the other hand, both APL_0421 or APJL_0446 were able to restore wild type AH-3 core when expressed into A. *hydrophila* AH-3Δ*wahF* mutant (Figure 4, lanes 10 and 11) but not APL_1403 and APJL_1428 (Figure 4, lanes 8 and 9), suggesting that the *A. pleuropneumoniae* heptosyltransferase III recognize the A. *hydrophila* AH-3Δ *wahF* mutant core [α-L,D-Hep-(1→6)-[α-L,D-Hep-(1→3]-α-L,D-Hep-(1→5)-Kdo-P] [26]. Thus, these results unambiguously allow assigning heptosyltransferase II and III functions to APL_1403 (APJL_1428) and APL_0421 (APJL_0446), respectively.

### WahD

**Figure 5:**
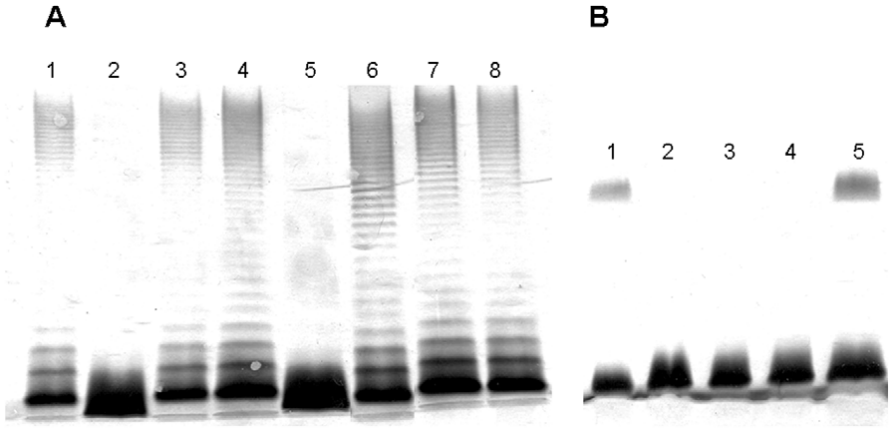
LPS analysis by SDS-PAGE. (**A**) LPS samples from *A. hydrophila* AH-3 (lane 1), AH-3Δ*wahD* (lane 2), AH-3Δ*wahD* (pBAD33-Gm-APL_0979) (lane 3), AH-3Δ*wahD* (pBAD33-Gm-APJL_0999) (lane 4), AH-3Δ*wahD* (pBAD33-Gm-PM1114) (lane 5), AH-3Δ*wahC* (lane 6), AH-3Δ*wahC* (pBAD33-Gm-APL_0981) (lane 7), and AH-3Δ*wahC* (pBAD33-Gm-APJL_1001) (lane 8). (**B**) LPS samples from *A. salmonicida* A450 (lane 1), A450Δ*wasD* (lane 2), A450Δ*wasD* (pBAD33-Gm-APL_0979) (lane 3), A450Δ*wasD* (pBAD33-Gm-APJL_0999) (lane 4), and A450 Δ*wasD* (pBAD33-Gm-PM1114) (lane 5).

As mentioned above, the WahD from *A. hydrophila* AH-3 is involved in the transfer of the D,D-Hep residue linked to Glc by an α 1,6 linkage while WasD from *A. salmonicida* transfer the L,D-Hep to Glc using the same linkage (Figure 1) [25], [33]. As seen in Figure 2 these proteins cluster together and APL_0979 and APJL_0999 are more similar to *Pasteurella multocida* PM1144 than to *Aeromonas* WahD or WasD. It is difficult to infer the function solely from the protein clustering because the only expected difference between WahD and WasD homologues should be in the region recognizing either ADP-D,D,Hep or ADP-L,D-Hep. From the known core LPS structures of *A. pleuropneumoniae* (Figure 1) it should be expected that both APL_0979 and APJL_0999 are homologues of WahD. If this hypothesis is correct, then expression of APL_0979 and APJL_0999 in *A. hydrophila* AH-3Δ*wahD* mutant should lead to the generation of full core LPS (Figure 5A, lanes 3 and 4). In addition, expression of APL_0979 and APJL_0999 in *A. salmonicida* A450Δ*wasD* mutant did not result in full core complementation (Figure 5B, lanes 3 and 4) while *P. multocida* PM1114 expressed in *A. salmonicida* A450Δ*wasD* did complement the mutant core LPS phenotype (Figure 5B, lane 5), while no such complementation was seen when it was expressed in *A. hydrophila* AH-3Δ*wahD* mutant (Figure 5A, lane 5).

To further confirm the function of APL_0979 and APJL_0999, LPS from *A. hydrophila* AH-3Δ*wahD*, *A. hydrophila* AH-3Δ*wahD* + pBAD33-Gm-APL_0979, and *A. hydrophila* AH-3Δ*wahD* + pBAD33-Gm-APJL_0999, were isolated. These LPS samples were hydrolyzed by mild-acid treatment and the core OS fraction isolated by chromatography. MS analysis of the OS fraction from *A. hydrophila* AH-3Δ*wahD* showed a major signal at *m*/*z* 1311.44 corresponding to a core OS (Hex_1_HexN_1_Hep_4_Kdo_1_) consistent with the loss of the outer-core trisaccharide fragment α-D,D-Hep-(1→6)-[β-D-Gal-(1→4)]-α-D,D-Hep from the full core LPS (Table 1). By contrast, MS analysis of the OS fraction from *A. hydrophila* AH-3Δ*wahD* + pBAD33-Gm-APJL_0999 showed a major signal at *m*/*z* 1857.617 (Table 1) in agreement with a full length AH-3 core (Hex_2_HexN_1_Hep_6_Kdo_1_) (Table 1) [25]. The second major signal at *m/z* 1503.498 was assigned to a core OS devoid of the terminal D,D-Hep and D-Gal from the wild type AH-3 core OS (Table 1). Similar results were obtained when analyzing the OS fraction from *A. hydrophila* AH-3Δ*wahD* + pBAD33-Gm-APL_0979 (data not shown). Taken together, these data allow to attribute a heptosyltransferase function involved in the transfer of the first D,D-Hep residue of the outer-core in *A. pleuropneumoniae* to APL-0979, and APJL_0999.

**Table 1:** Negative ion charge deconvoluted electrospray ionization mass spectral data of the core oligosaccharides released by mild acid hydrolysis from LPS of *A. hydrohila* AH-3Δ*wahD*, *A. hydrohila* AH-3Δ*wahD* (PBAD33-Gm-APJL_0999), A. *hydrohila* AH-3Δ*wahC*, and *A. hydrohila* AH-3Δ*wahC* (pBAD33-Gm-APJL_1001).

Strain	Molecular mass (Da)		Proposed composition
	observed	calculated^a^	
AH-3Δ*wahD*	1311.44	1311.43	anh-Kdo, 4 Hep, Hex, HexN
AH-3Δ*wahD* +APJL_ 0999	1937.59	1937.58	anh-Kdo, 6 Hep, 2 Hex, HexN, P
	1857.62	1857.61	anh-Kdo, 6 Hep, 2 Hex, HexN
	1695.57	1695.56	anh-Kdo, 6 Hep, Hex, HexN
	1503.50	1503.50	anh-Kdo, 5 Hep, Hex, HexN
AH-3Δ*wahC*	1665.58	1665.55	anh-Kdo, 5 Hep, 2 Hex, HexN
	1503.53	1503.50	anh-Kdo 5 Hep, Hex, HexN
	1311.46	1311.43	anh-Kdo, 4 Hep, Hex, HexN
AH-3Δ*wahC* + APJL_1001	1937.60	1937.58	anh-Kdo, 6 Hep, 2 Hex, HexN, P
	1857.63	1857.61	anh-Kdo, 6 Hep, 2 Hex, HexN
	1695.57	1695.56	anh-Kdo, 6 Hep, Hex, HexN
	1503.50	1503.50	anh-Kdo, 5 Hep, Hex, HexN

a Average mass units (in daltons) used for calculation of molecular masses based on the proposed composition, as follows: anh-Kdo, 202.048; Hep, 192.063; Hex, 162.053; HexN, 161.069; and P, 79.966.

### WahC

The *A. hydrophila* AH-3 WahC is responsible for the transfer of the terminal D,D-Hep residue of the AH-3 core LPS [25], and shows significant similarity to *A. pleuropneumoniae* APL_0981 and APJL_1001. No homologous protein from *A. salmonicida* was detected (Figure 2) in agreement with the presence of terminal D,D-Hep in *A. hydrophila* but not in *A. salmonicida* (Figure 1). A major difference between these two groups of proteins is that while the AH-3 and ATCC 7966 WahC are assigned to the GT9 family in the CAZY classification of glycosyltransferases, the two *A. pleuropneumoniae* proteins are actually grouped as non-classified glycosyltransferases on the basis of weak similarity to established glycosyltransferase families. However, in the Kegg database, both of these proteins are shown as having a glycosyltransferase family 9 (heptosyltransferase) motif. On the basis of the presence of a terminal D,D-Hep in the *A. pleuropneumoniae* core OS of serotype 2, 5a, and 5b (genome strain L20) [22], we hypothesized that APL_0981 would be a WahC homologue. Furthermore, APL-0981 is identical to APP7_1037 (serotype 7), and shows only one amino acid residue different when compared with APJL_1001 (serotype 3) for which the core LPS structure remains unknown.

To test the above hypothesis APL_0981 and APJL_1001 were introduced and expressed into *A. hydrophila* AH-3Δ*wahC*. As previously shown, the LPS of the AH-3 mutant still contains O-antigen (Figure 5, lane 6) and its core OS was devoid of the terminal D,D-Hep residue as demonstrated by MS analysis of its core OS fraction (Table 1) and methylation analysis [25]. *A. hydrophila* AH-3Δ*wahC* harboring pBAD33-Gm-APL_0981 or APJL_1001 recover full length core LPS since no differences in mobility of the lipidA-core band could be detected among LPS isolated from these strains and wild type AH-3 (Figure 5, lanes 6, 7, and 8). Furthermore, MS analysis of the core LPS fraction isolated from *A. hydrophila* AH-3Δ*wahC* harboring pBAD33-Gm-APJL_1001 gave a major signal a *m/z* 1857.63 corresponding to full length core OS (Hex_2_HexN_1_Hep_6_Kdo_1_), other signals were attributed to Hex_1_HexN_1_Hep_6_Kdo_1_ (*m/z* 1695.573) and Hex_1_HexN_1_Hep_5_Kdo_1_ (*m/z* 1503.495) (Table 1). Similar results were obtained with the core OS fraction from *A. hydrophila*

AH-3Δ*wahC* harboring pBAD33-Gm-APL_0981 (data not shown). These results, strongly suggests that *A. pleuropneumoniae* APL_0981 and APJL_1001 are functional homologues of *A. hydrophila* WahD and are responsible for the transfer of the terminal outer-core D,D-Hep residue (Figure 1).

## Discussion

The bioinformatic and phylogenetic analysis reported here shows that four L,D-heptosyltransferases (WaaC, OpsX, WaaF, and WaaQ) can be grouped on the basis of their amino acid similarity (Figure 2 and Table 2), while the heptosyltransferase WahE would constitute a more distant group. The two other L,D-heptosyltransferases WahF and WasD (ASA 4222) appear to be related to D,D-heptosyltransferases (WahD and WahC) (see cladogram in Figure 2). It is not clear that differences between residues found to be important in WaaC with those in other heptosyltransferase are specifically significant for the catalytic function and interaction with ADP-L,D-Hep of *E. coli* heptosyltranferase I (WaaC) [35]. The key residues for the catalytic function and interaction with ADP- L,D-Hep were D^13^, K^192^, E^222^, D^261^, and H^266^, the last two (D^261^and H^266^) present in the proposed heptosylytansferases signature sequence D^261^(T/S)(A/G)XXH^266^. These conserved residues were shown to interact with ADP-2-deoxy-2-fluoro-heptose [35]. The only residues that were found conserved in all the heptosyltransferases analyzed in this study (Table 2) were D^13^ and H^266^. The K^192^ was found to interact with the O5 and O6 atoms of the heptose, and it was present in L,D- heptosyltransferases WaaC, OpsX, WaaF, and WaaQ. It has been suggested that in D,D-heptosyltransferases the K^192^ could be substituted by other residues as by R in *Helicobacter pylori* [35], [36]. This is also the case for D,D-heptosyltransferases WahD and WahC where K^192^ is substituted by R or N or R, respectively. We also found K^192^ in all the analyzed L,D-heptosyltransferases except WahF with N or T substituting K^192^ (Table 2). The *E. coli* WaaC E^222^ residue, which interacts with the O2 and O3 residues of the heptose [35], was found in heptosyltransferases WahE, WahF, and WahD, being substituted by D in WaaQ and by other residues in OpsX, WaaF, WasD, and WahC (Table 2). *E. coli* WaaC D^261^ and H^266^ residues were previously shown to interact with O3 of the heptose and in addition H^266^ contacts with the 2’’ fluorine of the ADP-2-deoxy-2-fluoro-heptose [35]. They are found in all the heptosyltransferases analyzed in this work with the exception of WahF where D^261^ is substituted by S (Table 2). The WahF signature [S(T/S)(G/A)XXH] is unique because of the change in the heptosyltransferases consensus signature sequence [D(T/S)(G/A)XXH]. Minor differences were also observed for the signature sequences of D,D-heptosyltransferases WahD [D(T/S)(S/A)XXH] and WahC [D(T/G)(G/S)XXH]. All these results are summarized in Table 2.

**Table 2:** The presence of *E. coli* WaaC enzyme key residues for the catalytic function and interaction with ADP- L,D-Hep [35] in well characterized heptosyltransferases in different Gram-negative bacteria (*Enterobacteriaceae* and *Aeromonadaceae*). Comparison of the identified *E. coli* WaaC enzyme key residues based in amino acid alignment.

Enzyme	Linkage	Amino acid position^a^								
		13	192	222	261	262	263	264	265	266
WaaC	α-LD-HepI-(1→5)-Kdo	D	K	E	D	T/S	G/A	X	X	H
OpsX	α-LD-HepI-(1→5)-Kdo(4P)	D	K	L/Y/R	D	T/S	G/A	X	X	H
WaaF	α-LD-HepII-(1→3)-α-LD-HepI	D	K	K/H/V/S	D	T/S	G/A	X	X	H
WahE	α-LD-HepIV-(1→6)-α-LD-HepI	D	K	E	D	T/S	G/A	X	X	H
WaaQ	α-LD-HepIII-(1→7)-α-LD-HepII	D	K	D	D	T/S	G/A	X	X	H
WahF	α-LD-HepIII-(1→2)-α-LD-HepII	D	N/T	E	S	T/S	G/A	X	X	H
WasD	α-LD-HepV-(1→6)-β-D-Glc	D	K	E/A	D	T/S	S/A	X	X	H
WahD	α-DD-HepV-(1→6)-β-D-Glc	D	R	E	D	T/S	S/A	X	X	H
WahC	α-DD-Hep-(1→6)-α-DD-Hep	D	N/R	K/P	D	T/G	G/S	X	X	H

a Amino acid position referred to *E. coli* K12 [35].

This analysis stresses the importance of experimental identification of the function of hypothetical heptosyltransferases. Two approaches are possible; one is based on the construction of individual mutations and analysis of the core LPS defects [25]; and the second one, uses surrogate LPS molecules to test the function of individual heptosyltransferases. The first approach can be difficult in some bacterial species and has the problem that the conclusions are based on a negative feature represented by the shortage of the wild type core, in addition sometimes the interpretation of data is complicated by the presence of different core OS in the mutant strains. The second approach requires having surrogate LPS molecules highly similar to the predicted heptosyltransferase LPS acceptor molecule. The LPS surrogate acceptors would be provided by previously obtained mutants from other bacteria, where the acceptor (mutant strains) should be constructed in such a way that there are no downstream effects. The use of surrogate LPS molecules allows for a positive trait: the addition of a new residue to surrogate core LPS, and if adequate the analysis is facilitated because the modified surrogate core LPS will acquire also the corresponding O-antigen domain (Figures 3, 4, and 5) and further confirmed by composition and MS analysis (Table 1).

In this work we show the feasibility of this approach by identifying the *A. pleuropneumoniae* heptosyltransferases from strains L20 and JL03. The same functions are expected from the homologous proteins from strain AP76 (Figure 2). Furthermore, our results based in the use of surrogate LPS acceptor from an *A. hydrophila* Δ*wahC* mutant (Figure 5, lanes 6, 7, and 8) and MS analysis (Table 1) allow to establish unambiguously an heptosyltransferase function for APL_0981 and APJL_1001 although these two proteins are not included in the glycosyltransferase GT9 family in the CAZY classification. These two proteins are involved in the addition of the terminal D,D-Hep residue of the *A. pleuropneumoniae* core LPS (Figure 1), and then besides the sequence similarity should be included as GT9 family members.

There is an increasing interest in the development of antivirulence agents and one of the potentially interesting targets for them is the WaaC heptosyltransferase I [37]. Our and previous analyses of the heptosyltransferases involved in LPS biosynthesis clearly show that the most conserved region corresponds to the residues involved in catalytic function and interaction with the ADP-L,D-Hep. Thus, the search for antivirulent inhibitors of the WaaC and other heptosyltransferases interacting with these residues appears be the best way to proceed.

## Materials and Methods

### Bacterial strains, plasmids, and growth conditions

Bacterial strains, and plasmids used in this study are listed in Table 3. *Aeromonas* strains were routinely grown on tryptic soy broth (TSB) or tryptic soy agar (TSA) at 30°C unless stated otherwise. *A. pleuropneumoniae* strains were grown on Brain Herat Infusion (BHI) at 37°C and *E. coli* strains were grown on Luria-Bertani (LB) Miller broth and LB Miller agar at 37°C [38]. Kanamycin (50 µg/ml), rifampicin (100 µg/ml), or gentamycin (20 µg/ml) were added to the different media.

**Table 3:** Bacterial strains and plasmids used.

Strain or plasmid	Relevant characteristics	Reference or source
Strains		
*A. pleuropneumoniae*		
L20	Wild-type, capsular serotype 5b	U.A.B.^a^
JL03	Wild.type, capsular serotype 3	U.A.B.
*E. coli*		
LMG194	F^-^ Δ*lacX74 galE galK thi rpsL* Δ*phoA* (PvuII) Δ*ara714 leu*::Tn*10*	[48]
HB101	F^-^ *mcrB mrr hsdS20* (r_B_^-^ m_B_^-^) *recA13 leuB6 ara-14 proA2 lacY1 galK2 xyl-5 mtl-1 rpsL20* (Sm^R^) *glnV44* λ^-^	[49]
*K. pneumoniae*		
52145Δ*waaC*	In frame deletion *waaC* mutant	[34]
*A. hydrophila*		
AH-3	Wild-type, O34	[50]
AH-3Δ*wahD*	*wahD* Km::Tn5; Rif^R^ Km^R^	[25] b
AH-Δ*opsX*	*opsX* (*waaC* a) Km::Tn5; Rif^R^ Km^R^	[25]
AH-3Δ*wahC*	In frame deletion mutant Δ*wahC*	[25]
AH-3Δ*waaF*	In frame deletion mutant Δ*waaF*	[25]
AH-3Δ*wahF*	*wahF* Km::Tn5; Rif^R^ Km^R^ insertion mutant	[25]
*A. salmonicida*		
A450		[33]
A450Δ*wasD*	*wasD* (*wahD*-like^a^) Km::Tn5; Rif^R^ Km^R^ insertion mutant	[33]
Plasmids		
pRK2073	Conjugation helper plasmid, Spc^R^	[51]
pBAD33-Gm	Arabinose-inducible expression vector, Gm^R^	[33]
pBAD33-Gm-APL_0186	pBAD33-Gm with APL_0186 from strain L20	This study
pBAD33-Gm-APL_0421	pBAD33-Gm with APL_0421 from strain L20	This study
pBAD33-Gm-APL_0979	pBAD33-Gm with APL_0979 from strain L20	This study
pBAD33-Gm-APL_0981	pBAD33-Gm with APL_0981 from strain L20	This study
pBAD33-Gm-APL_1402	pBAD33-Gm with APL_1402 from strain L20	This study
pBAD33-Gm-APL_1403	pBAD33-Gm with APL_1403 from strain L20	This study
pBAD33-Gm-APJL_0187	pBAD33-Gm with APJL_0187 from strain JL03	This study
pBAD33-Gm-APJL_0446	pBAD33-Gm with APJL_0446 from strain JL03	This study
pBAD33-Gm-APJL_0999	pBAD33-Gm with APJL_0999 from strain JL03	This study
pBAD33-Gm-APJL_1001	pBAD33-Gm with APJL_1001 from strain JL03	This study
pBAD33-Gm-APJL_1427	pBAD33-Gm with APJL_1427 from strain JL03	This study
pBAD33-Gm-APJL_1428	pBAD33-Gm with APJL_1428 from strain JL03	This study

a Universidad Autónoma Barcelona, Spain

b Original nomenclature [25]

### General DNA methods and computer analysis of sequence data

General DNA manipulations were done essentially as described [39]. DNA restriction endonucleases, T4 DNA ligase, *E. coli* DNA polymerase (Klenow fragment), and alkaline phosphatase were used as recommended by the suppliers. Deduced amino acid sequences used from different *Enterobacteriaceae* and *Aeromonadaceae* were obtained from the GenBank, EMBL, and SwissProt databases. ClustalW [40] was used for multiple-sequence alignments with a gap open penalty of 10, gap extension penalty of 0.05, no weight transition, with hydrophilic gaps and GPSNDQERK hydrophilic residues for proteins, and BLOSUM as selected weight matrix.

### Plasmid constructions and mutant complementation studies

For complementation studies, the *A. pleuropneumoniae* genes (APL_0186, APL_0421, APL_0979, APL_0981, APL_1402, APL_1403, APJL_0187, APJL_0446, APJL_0999, APJL_1001, APJL_1427, and APJL_1428) were PCR-amplified by using specific primer pairs (Table 4) and ligated to the plasmid pBAD33-Gm. The plasmid constructions were transformed into *E. coli* LMG194 by electroporation, plated on gentamycin LB agar plates and incubated at 37°C. Plasmids with the amplified genes were independently transferred into the corresponding mutants by triparental mating using the mobilizing strain HB101/pRK2073. Transconjugants were selected on plates containing gentamycin and rifampicin and confirmed by PCR. Each gene was expressed from the arabinose-inducible and glucose-repressible pBAD33 promoter. Repression from the *araC* promoter was achieved by growth in medium containing 0.2% (w/v) D-glucose, and induction was obtained by adding l-arabinose to a final concentration of 0.2% (w/v). The cultures were grown for 18 h at 30°C in TSB medium supplemented with gentamycin and 0.2% glucose, diluted 1∶100 in fresh medium (without glucose) and grown until they reached *A*_600 nm_ of about 0.2. Then l-arabinose was added, and the cultures were grown for another 2 h. Repressed controls were maintained in glucose-containing medium.

**Table 4:** Primers used to amplify and subclone individual genes in pBAD33-Gm.

Primersa,b	Amplified Fragment
*opsX* (APL_0186 and APJL_0187)	
AXf: 5’-TGCTCTAGATAATCAGACCGAACCCACGC-3’	
AXr: 5′-CCCAAGCTTCGGCCGGGGTGAGTAATA-3’	AXf-AXr (1243 bp)
*wahF* (APL_0421 and APJL_0446)	
AF2f: 5’-TGCTCTAGATACCGAGCAAGCGGTCAAAT-3’	
AF2r: 5’-CCCAAGCTTGACTTGCCATTTTAACAGGCTTT-3’	AF2f-AF2r (1185 bp)
*wahD* (APL_0979 and APJL_0999)	
ADf: 5’-TGCTCTAGATTAGTCCCGGCTCGGAAATC-3’	
ADr: 5’-*CTGCAG*TGCCCCAGCTCTTGTTCAAT-3’	ADf-ADr (1219 bp)
*wahC* (APL_0981 and APJL_1001)	
AC2f: 5’-TGCTCTAGATGTGCGCATGGATTTTACGG-3’	
AC2r: 5’-*CTGCAG*TGTCCTGGATTCAAGCGGTC-3’	AC2f-AC2r (1155 bp)
*waaC* (APL_1402)	
AC11f: C: 5’-TGCTCTAGACGACCGCTTGTTCGTATTCAT-3’	
AC11r: D: 5’-CCCAAGCTTGCAAGCCTCTAATGCAGGAAC-3’	AC11f-AC11r (1198 bp)
*waaC* (APJL_1427)	
AC12f: 5’-TGCTCTAGATGCAGGTTTAGACCGCTTG-3’	
AC12r: 5’-CCCAAGCTTTCACACGCGGTTTGCG-3’	AC12f-AC12r (1167 bp)
*waaF* (APL_1403 and APJL_1428)	
AF1f: 5’-TGCTCTAGATGACCGCTTGTTATTTTAGGGA-3’	
AF1r: 5’-*CTGCAG*TGACCGCTTGCAATTAGCCT-3’	AF1f-AF1r (1082 bp)

a Bold, underlined, and italic letters *Xba*I, *Hind*III, and *Pst*I restriction sites, respectively.

b The PCR amplified products were digested with the indicated restriction enzymes and ligated to *Xba*I-*Hind*III or *Xba*I-*Pst*I digested pBAD33-Gm.

### LPS isolation and SDS-PAGE

For screening purposes LPS was obtained after proteinase K digestion of whole cells [41]. LPS samples were separated by SDS-PAGE or N-[2-hydroxy-1,1-bis(hydroxymethyl)ethyl]glycine (Tricine)-SDS-PAGE [42], [43] and visualized by silver staining as previously described [41], [44]. For large-scale isolation LPS was extracted from cells grown in TSB at 30°C. Cells were dried and suspended in 25 mM Tris-HCl buffer containing 2 mM CaCl_2_ pH 7.63 (10 mL g ^-1^) were treated at 37°C with RNAse, DNAse (24 h, 1 mg/g each), and then with Proteinase K (36 h, 1 mg/g). The suspension was dialyzed and lyophilized. The phenol/chloroform/light petroleum ether method [45] was used for strains producing rough LPS, while the phenol/water procedure [46] was used for the strains producing the O antigen domain (smooth LPS).

### Preparation of oligosaccharide fraction

A portion of the LPS (∼50 mg) from each strain was heated with aqueous 2% HOAc (6 mL) at 100 °C for 45 min. The precipitate was removed by centrifugation (13,000 *g*, 20 min) and the supernatant fractionated on a column (56×2.6 cm) of Sephadex G-50 (S) in 0.05 M pyridinium acetate buffer pH 4.5 with monitoring using a differential refractometer (Knauer, Germany).

### Mass spectrometry

Electrospray ionization mass spectra were run in the negative ion mode using a micrOTOF II instrument (Bruker Daltonics, USA). Mass spectra were acquired using standard experimental sequences as provided by the manufacturer. Samples (∼50 ng µL^-1^) were dissolved in a 1∶1 (v/v) water-acetonitrile mixture and sprayed at a flow rate of 3 µL min^-1^. Capillary entrance voltage was set to 4.5 kV and exit voltage to −150 V; drying gas temperature was 180°C. The spectra showing several charge states for each component were charge deconvoluted, and mass numbers given refer to monoisotopic molecular masses.

### Phylogenetic analysis

The analysis was performed by running the phylogenetic inference software Protpars from the PHYLIP (Phylogeny Inference Package) package version 3.5c (Department of Genetics University of Washington, Seattle) [47], using heptosyltransferases whose function is experimentally proved from enteric bacteria, *Aeromonas* or *Pasteurella multocida* with those of *A. pleuropneumoniae* strains L20 (serotype 5b) [26], JL03 (serotype 3) [27], and AP76 (serotype 7) [28]. The cladogram is indicative by the separation and the large of the lanes from the values obtained running the program (the scale bar indicates an evolutionary distance of 0.1 aminoacid substitutions per position).
